# Physicians’ adherence to management guidelines for *H. pylori* infection and gastroesophageal reflux disease: a cross-sectional study

**DOI:** 10.1186/s13584-020-00389-y

**Published:** 2020-06-11

**Authors:** Wasef Na’amnih, Amir Ben Tov, Amna Bdair-Amsha, Shlomi Cohen, Judith Tsamir, Gabriel Chodick, Khitam Muhsen

**Affiliations:** 1 Department of Epidemiology and Preventive Medicine, School of Public Health, Sackler Faculty of Medicine, Tel Aviv University, 6997801 Tel Aviv, Israel; 2 Maccabi Healthcare Services, Tel Aviv, Israel; 3 Pediatric Gastroenterology Unit, “Dana-Dwek” Children’s Hospital, Tel Aviv Sourasky Medical Center, Tel Aviv, Israel

**Keywords:** Survey, *Helicobacter pylori*, Gastroesophageal reflux disease, Guidelines, Primary care physicians

## Abstract

**Background:**

Primary care physicians (PCPs) play a pivotal role in the management of illnesses of the digestive tract. The study aim was to assess the adherence of PCPs to the guidelines on the management of *Helicobacter pylori* (*H. pylori*) infection and gastroesophageal reflux disease (GERD) in adults.

**Methods:**

We conducted a cross-sectional study during March–July 2017 using the survey platform of Maccabi Healthcare Services in Israel. The study questionnaire assessed adherence to the Maastricht/Florence guidelines on *H. pylori* infection and the American College of Gastroenterology guidelines on the management of GERD. We sent the study questionnaires to a random sample of 610 PCPs via electronic mails. We contacted those who did not respond by telephone; eventually 180 physicians completed the survey.

**Results:**

Ninety (50%) and 60 (36%) of the responders reported using professional guidelines for the diagnosis and management of *H. pylori* infection and GERD, respectively. Of the 180 participants, 153 (85%) reported referring patients with suspected peptic ulcer disease to *H. pylori* testing, 109 (61%) reported referring patients with unexplained iron deficiency anemia and 83 (46%) refer relatives of gastric cancer patients. In caring for young patients who have dyspepsia without alarm symptoms, 127 (74%) reported referral to a urea breath test for the diagnosis of *H. pylori* infection, and 136 (81%) referral to a specialist in gastroenterology if alarm symptoms present. Triple therapy with proton pump inhibitors/clarithromycin/amoxicillin or metronidazole was reported as first-line therapy by 141 (83%) participants. For GERD, 94–98% of the participants followed the appropriate recommendations.

**Conclusions:**

We identified gaps between the practices of PCPs and the guidelines on *H. pylori* infection management**,** while guidelines on GERD management are well adopted. Simplification of the guidelines and exploring barriers towards their implementation by PCPs is warranted.

## Background

Gastrointestinal complaints of the upper digestive tract such as abdominal pain, heartburn, nausea and vomiting are common in the primary care setting, while the differential diagnosis might vary from functional disorders to malignancy [1–4].

The diagnosis and management of gastroesophageal reflux disease (GERD) and peptic ulcer disease are of particular interest. *Helicobacter pylori* (*H. pylori*) infection is the main cause of peptic ulcer disease and of gastric cancer [5]. According to the Maastricht V/Florence Consensus Report on the management of *H. pylori* infection, in young patients with uninvestigated dyspepsia the ‘test-and-treat’ strategy with non-invasive test, usually urea breath test (UBT) is recommended. In older adults and in patients with alarm symptoms such as weight loss, gastrointestinal bleeding, it is recommended to perform oesophago-gastro-duodenoscopy. If *H. pylori* is identified, a-14 day treatment is recommended, using proton pump inhibitors (PPIs) with clarithromycin, amoxicillin or metronidazole, with or without bismuth. At least four weeks after completing therapy, a non-invasive test is recommended to confirm eradication of the infection [6].

The prevalence of GERD has increased over the past few years [7, 8]. GERD causes substantial burden to the health care system. In patients with typical GERD symptoms (e.g., heartburn and regurgitation), empiric PPI therapy is a reasonable approach to confirm GERD diagnosis, while in patients with alarm symptoms, endoscopy should be performed [9].

Primary care physicians (PCPs) play a pivotal role in the management of GERD and *H. pylori* infection. Deviations from guidelines for managing *H. pylori* infection and GERD were reported, including in indications for testing, choosing diagnostic tests, treatment and follow-up [10–21]. Studies from Israel demonstrated gaps in the adherence to guidelines for the management of *H. pylori* [22, 23] and GERD [24] especially among PCPs. In a large database analysis of Maccabi Health Services (MHS) we identified variations in the use of diagnostic tests of GERD compared to the guidelines [25].

The current period is characterized by high accessibility to online resources, and by the repercussions of the “choosing wisely” initiative [26]. This warrants an updated assessment of adherence of PCPs to the guidelines on management of *H. pylori* infection compared to GERD. The aim of this study was to assess the adherence of PCPs to guidelines on the management of *H. pylori* infection and GERD in adults.

## Methods

### Study design and population

We conducted a cross-sectional study between March and July 2017 using the survey platform of MHS, the second largest health maintenance organization (HMO) in Israel. In Israel, access to care is universal to all citizens, according to the National Health Insurance Law, implemented since 1995. Most services are given at no cost at point of care. Citizens should be insured in one of the four HMOs [27, 28]. MHS currently has over two million members, comprising about 25% of Israel’s population.

### Data collection

The study team constructed the study questionnaire (see Additional file 1). The questions accessed information on the management of *H. pylori* infection and GERD, and physicians’ referral to diagnostic tests, prescriptions for treatment of these conditions. In several questions, we asked the physicians to rank the frequency that selected clinical scenarios occurred at their practices. The replies were according to a Likert scale: always, usually yes, usually no and never. In analysis of the data, the replies “always and usually yes” were combined as “yes” and the replies “usually no” and “never” were combined as “no”.

The 2012 Maastricht IV/Florence guidelines on the management of *H. pylori* infection [29] and the 2013 American College of Gastroenterology (ACG) guidelines on the management of GERD [9] were considered as references in this study, since they were the most updated guidelines during the study period.

Information accessed from the MHS database included characteristics of the physicians, such as age (in years), sex, the year they began working at MHS and their type of work relationship with MHS (self-employed vs. employee). Information on the number of years since the board certification was obtained via the questionnaire. The survey questionnaire was distributed to physicians through the electronic mail system of MHS. The message was sent on two occasions, three to four weeks apart, to increase the response rate. Additionally, the study team contacted by telephone physicians who did not open the survey link, and interviewed those who agreed to participate in the study. Overall, 610 PCPs were randomly selected. Of these, 183 physicians responded; three physicians, who started the questionnaire, did not complete it. Thus 180 physicians were included in the study (i.e. a response rate of 30%).

### Statistical analysis

Differences between responders and non-responders in background characteristics were compared using the chi-square test for categorical variables and the Student’s *t* test for continuous variables. The study sample was described using frequencies and percentages for categorical variables, and means and standard deviation (SD) for continuous variables. We performed unweighted and weighted analyses. The weights were determined using the inverse probability weighting method [30], The probability to participate in the study was obtained from a multivariable logistic regression model in which the dependent variable was participation in the study (coded as 1 = yes and 0 = no) and the independent variables were age, sex and the year of starting work at MHS.

Differences in the characteristics of participants who did and did not follow recommendations regarding referral for *H. pylori* testing were examined using the Student’s *t* test for continuous variables and the chi square test and Fisher Exact test for categorical variables. Statistical significance was determined as *p* < 0.05. The Benjamini and Hochberg false discovery rate method was used to adjust for multiple comparisons [31]. We analysed the data using SPSS version 25 (IBM, New York, United States).

## Results

Characteristics of physicians who participated in the study are presented in Table 1.

**Table 1:** Characteristics of the respondents and non-respondents to the questionnaire

	Primary care physician		***P***
	Respondents ***N*** = 171*	Non-respondents ***N*** = 429*	0.3
Age, mean (SD), years	53.6 (12.0)	54.6 (10.7)	0.09
Sex, males	104 (61%)	229 (53%)	0.02
Started to work at MHS from 2010 onward	67 (39%)	127 (30%)	0.7
Employment type, self-employed	147 (86%)	359 (84%)	0.3

* Missing data: Nine respondents; one non-respondent

MHS: Maccabi Healthcare Services; SD: standard deviation

### Practices of PCPs regarding the management of *H. pylori* infection

Ninety (50%) participants reported utilization of any guidelines for the management of *H. pylori* infection; of them, 82 specified which guidelines: 35% reported using the Israeli gastroenterology guidelines, 8% American Gastroenterological Association guidelines, 5% the Maastricht guidelines, 31% reported using the UpToDate website [32], 21% relied on other resources.

#### Referral for a diagnostic test of *H. pylori* infection and treatment of the infection

Eighty-five percent of participants reported referring patients with suspected gastric or duodenal ulcer to a diagnosis of *H. pylori* infection. Referrals to *H. pylori* testing in first-degree relatives of gastric cancer patients and unexplained iron deficiency anemia (IDA) were reported by 46 and 61% of the participants, respectively (Table 2).

**Table 2:** Self-reported practices of primary-care physicians in the management of *H. pylori* infection in adults

	Number/Total (percent)	Weighted Percent*
**Refer for*H. pylori*diagnosis in the case of** **		
Suspected duodenal or gastric ulcer	153/180 (85%)	84%
First degree relatives with gastric cancer	83/180 (46%)	47%
Unexplained iron deficiency anemia	109/180 (61%)	59%
Before starting long-term use of aspirin or NSAIDs in patients with a history of peptic disease	82/180 (46%)	44%
**Prescription of first-line therapy**		
Triple therapy with PPIs/clarithromycin/amoxicillin or metronidazole	141/171 (83%)	82%
Quadruple therapy based on Bismuth	15/171 (9%)	9%
Quadruple therapy non-Bismuth	7/171 (4%)	4%
Refer to gastroenterologist	2/171 (1%)	2%
Other	6/171 (3%)	3%
**Duration of treatment**		
7 days	15/171 (9%)	8%
10 days	83/171 (48%)	51%
14 days	65/171 (38%)	36%
Other	8/171 (5%)	5%
**Follow-up test**		
UBT at least 1 month after therapy	95/171 (56%)	57%
Refer to specialist in gastroenterology	4/171 (2%)	1%
Stool antigen detection EIA at least 1 month after therapy	2/171 (1%)	1%
Serology at least 1 month after therapy	5/171 (3%)	2%
Do not refer to a test if symptoms resolve	58/171 (34%)	34%
Other	7/171 (4%)	5%
**In case of treatment failure**		
Refer to a specialist in gastroenterology	74/171 (43%)	45%
Do not refer to a test if symptoms resolve	43/171 (25%)	23%
Recommend the same treatment for a longer duration	5/171 (3%)	2%
Recommend a different treatment	49/171 (29%)	30%

* Percentage obtained by inverse probability weighting

** Physicians who answered “always” or “usually”

EIA: Enzyme immunoassay; NSAID: non-steroidal anti-inflammatory drugs; PPIs: proton pump inhibitors; UBT: Urea breath test

In evaluating young patients with dyspepsia without alarm symptoms, most (74%) participants reported referral to the UBT as the main diagnostic test, 6% reported using the stool antigen detection enzyme immunoassay (EIA), 10% referred these patients to gastroscopy and to specialists in gastroenterology, and 10% to other tests. In evaluating patients with alarm symptoms, most (81%) participants reported that they usually refer to a specialist in gastroenterology; gastroscopy, UBT, the stool antigen detection EIA, and other tests were reported less frequently.

Prescribing triple therapy as first-line treatment was reported by most participants.

The weighted analysis yielded similar results (Table 2).

### Factors related to PCPs’ practices regarding *H. pylori* infection testing

Participants who referred for *H. pylori* infection diagnosis in suspected duodenal or gastric ulcer were younger than those who did not refer (*p* = 0.02), as well as those who referred to UBT/stool antigen EIA in test-and-treat strategy vs. those who did not follow these recommendations (*p* = 0.04). The participants who followed the recommendations of testing *H. pylori* in patients with unexplained IDA, and referral to UBT/stool antigen EIA in test-and-treat strategy had more recently underwent board certification than had those who did not follow these recommendations (p = 0.02 and *p* = 0.006, respectively). Compared to those who did not follow these guidelines, a higher proportion of those who followed them began working in MHS in 2010 or later. Participants who agreed with the statement that *“H. pylori* is a definitive cause of gastric cancer” were more likely to refer patients with dyspepsia and alarm symptoms for endoscopy or to a gastroenterology specialist (*p* = 0.03) (Table 3). Adjustment for multiple comparisons yielded *p* = 0.09 for all these differences.

**Table 3:** Factors related to primary-care physicians’ referrals of adults for *H. pylori* infection testing

	Clinical characteristics of the patients referred for testing												Type of testing					
	Suspected duodenal or gastric ulcer			First-degree relatives of gastric cancer patients			Unexplained IDA			History of peptic disease, before long-term use of NSAIDs			UBT/stool antigen EIA in test-and-treat strategy			Endoscopy/specialist-alarm symptoms		
	Yes	No	***P***	Yes	No	***P***	Yes	No	***P***	Yes	No	***P***	Yes	No	***P***	Yes	No	***P***
Number	146	25		78	93		102	69		76	95		136	27		160	8	
Age, mean (SD)	52.6 (12.3)	59.2 (8.2)	0.02	55.0 (10.4)	52.4 (13.1)	0.3	52.7 (11.9)	54.8 (12.1)	0.2	53.3 (12.6)	53.8 (11.6)	0.8	52.5 (12.4)	58.2 (9.0)	0.04	53.9 (12.0)	52.0 (10.9)	0.6
Years since board certification, mean (SD)	19.2 (12.5)	24.1 (10.3)	0.1	19.7 (11.6)	20.0 (13.1)	0.7	18.0 (12.7)	22.8 (11.1)	0.02	19.9 (12.4)	19.9 (12.3)	0.9	18.5 (12.2)	5.4 (12.3)	0.006	19.6 (12.3)	24.3 (12.8)	0.3
Started to work at MHS 2010 onward, n (%)	62 (43)	5 (20)	0.03	28 (36)	39 (42)	0.4	47 (46)	20 (29)	0.03	30 (40)	37 (39)	0.9	56 (41)	6 (22)	0.06	58 (38)	2 (33)	0.8
*H. pylori* is a definitive cause of gastric cancer, n (%)	124 (84)	20 (77)	0.3	72 (91)	72 (77)	0.01	88 (86)	56 (79)	0.2	68 (84)	76 (83)	0.8	116 (84)	27 (80)	0.5	136 (85)	4 (50)	0.03

EIA: enzyme immunoassay; IDA: iron deficiency anemia; MHS: Maccabi Healthcare Services; NSAIDs: non-steroidal anti-inflammatory drugs UBT: urea breath test; SD: standard deviation

### Practices of PCPs regarding the management of GERD

Overall, 168 participants completed the GERD component of the survey; of them, 36% reported utilization of any guidelines for the management of GERD. Fifty-nine specified which guidelines: 37% reported using the Israeli gastroenterology guidelines, 8% the American/European Gastroenterology Association guidelines, 31% reported using the UpToDate website [33], 24% reported other resources.

Regarding GERD, most participants reported that good adherence with the recommendations (Table 4).

**Table 4:** Self-reported practices of primary-care physicians in the management of gastroesophageal reflux disease in adults (*N* = 168)

	Number (percent) *	Weighted Percent**
**I recommend performing barium radiographs as part of GERD work-up**	10 (6%)	5%
**If complaints of chest pain exist, I refer to cardiologic work-up before the diagnosis of GERD**	138 (82%)	82%
**I recommend empiric treatment with PPIs for patients with typical symptoms of uncomplicated GERD**	165 (98%)	99%
**I recommend continuing therapy with PPIs for patients with persistent symptoms after discontinuation of initial treatment**	134 (80%)	83%
**I recommend changes in diet and reducing products that might increase symptoms of GERD such as caffeine, chocolate and fried food**	157 (94%)	94%
**I recommend sleeping with the head of the bed elevated for patients with GERD**	139 (83%)	84%
**For patients with obesity and with non-complicated GERD, I recommend weight loss**	159 (95%)	94%

* Physicians who answered “always” or “usually” out of 168 responders to this part

** Inverse probability weighting

PPIs: proton pump inhibitors; GERD: gastroesophageal reflux disease

## Discussion

The main finding of this survey was the limited adherence of Israeli PCPs to the guidelines on the management of *H. pylori* infection, and their relatively high adherence to the guidelines on the management of GERD.

Referral to *H. pylori* testing was reported by 85% of the study participants in their investigations of peptic ulcer, despite strong recommendation to test and treat *H. pylori* infection in patients with this condition [6, 29]. About half of the participants reported referring first-degree relatives of gastric cancer patients to *H. pylori* testing. Current evidence suggests that eradication of *H. pylori* might reduce the risk of gastric cancer [34]. *H. pylori* is transmitted between family members during childhood [32, 35]. Concurrent *H. pylori* infection and a family history showed a synergetic additive effect on the risk of gastric cancer [36]. This accentuates the importance of *H. pylori* testing for individuals with a family member who had gastric cancer.

Testing and treating *H. pylori* infection are recommended for patients with unexplained IDA [6, 29]. However, this recommendation was shown to be only partially followed in the current study. This concurs with findings observed even among specialists in gastroenterology in the United States [19]. The low adherence to this recommendation might be explained by physicians’ skepticism regarding extra-gastric effects of *H. pylori* infection.

Most participants in our study reportedly prescribed triple therapy. This is despite the increase in clarithromycin resistance in *H. pylori* strains in Israel [37] and the low success rates in *H. pylori* eradication [38].

Collectively, our updated evidence reinforces findings from previous studies [10, 11, 13–15, 17] regarding gaps between clinical guidelines and practices of PCPs in the management of *H. pylori* infection in adults. Physicians who followed the recommendations in our study were younger; less time elapsed since their board certification and they started working at MHS more recently than did those who did not follow recommendations. These findings corroborate with previous evaluations that indicated reduced quality of care performance among physicians with increasing years in practice [39]. Various barriers of adherence to guidelines among physicians were shown. These included low awareness, familiarity and agreement with the guidelines; difficulty in overcoming the inertia of previous practices; and external barriers that inhibit the ability to perform the recommendations [40]. These factors should be taken into account when planning educational interventions aiming to increasing adherence with the guidelines.

In contrast to the findings regarding *H. pylori* management, the guidelines for GERD management were found to be well adopted by the participants, and consistent with the recommendation of the ACG guidelines [9]. Similar observations were reported from Eastern Asian countries [41] and Germany [42], while others [20, 21, 24] reported some gaps.

Our study has limitations. The response rate to participate was low, despite our efforts to increase compliance via repeated messages and phone calls. Nonetheless, responders and non-responders had similar demographic profiles.

## Conclusions

Adherence of PCPs to guidelines on the management of *H. pylori* infection in adults was sub-optimal, while adherence to the guidelines on GERD management was relatively satisfactory. Simplification of the guidelines and exploring barriers towards their implementation by PCPs is warranted.

## Supplementary information

**Additional file 1.** Study questionnaire.

## Data Availability

The data used to support the findings of this study have not been made available because of legal and ethical restrictions.

## Abbreviations

ACG: American College of Gastroenterology
EIA: Enzyme immunoassay
GERD: Gastroesophageal reflux disease
*H. pylori*: *Helicobacter pylori*
HMO: Health maintenance organization
IDA: Iron deficiency anemia
MHS: Maccabi Healthcare Services
NSAIDs: Non-steroidal anti-inflammatory drugs
PCPs: Primary care physicians
PPIs: Proton pump inhibitors
SD: Standard deviation
UBT: Urea breath test
