# Supplementary material for: Physicians’ adherence to management guidelines for H. pylori infection and gastroesophageal reflux disease: a cross-sectional study
Source: Isr J Health Policy Res. 2020 Jun 11;9:28. doi: 10.1186/s13584-020-00389-y (PMC7291643; doi:10.1186/s13584-020-00389-y)
Supplement: Supplementary file 1 — Additional file 1. Study questionnaire. [file 13584_2020_389_MOESM1_ESM.pdf]

Appendix 1- Questionnaire

Module I: The following questions are related to the diagnosis and treatment of *Helicobacter pylori* infection in adults. **Please, choose only one answer in all the following questions.**

1. Do you use any guidelines for the management of *H. pylori* infection?

1) No

2) Yes, please specify which guidelines: ____

2. In the following situations, how often do you usually refer to the diagnosis of *H. pylori*?

	Always	Usually, yes	Usually, no	Never
Suspected duodenal or gastric ulcer	4	3	2	1
First-degree relatives of gastric cancer patients	4	3	2	1
Gastroesophageal reflux disease	4	3	2	1
Unexplained iron deficiency anemia	4	3	2	1
History of peptic ulcer in patients who began Prolonged therapy of aspirin or NSAIDs	4	3	2	1

3. To what extent do you agree with the following statement: "*H. pylori* is a definitive cause of gastric cancer":

1) Strongly agree

2) Somewhat agree

3) Disagree

4) Strongly disagree

4. **To diagnose *H. pylori* in young patients who have dyspepsia without alarm symptoms such as weight loss, anemia, I usually refer to:**

1) Specialist in gastroenterology

2) Gastroscopy

3) Urea breath test

4) Stool antigen enzyme immunoassay

5) Other, please specify: ____________________

5. **To diagnose *H. pylori* in patients with dyspepsia and alarm symptoms such as weight loss, I usually refer to:**

1) Specialist in gastroenterology

2) Gastroscopy

3) Urea breath test

4) Stool antigen enzyme immunoassay

5) Other, please specify: ____________________

6. **What is the first-line treatment that you usually prescribe for patients with a positive *H. pylori* result?**

1) Triple therapy with PPIs /clarithromycin/amoxicillin or metronidazole

2) Quadruple based on Bismuth

3) Quadruple with PPIs /clarithromycin/amoxicillin/metronidazole

4) Probiotics

5) Refer the patient to a specialist in gastroenterology

6) Other; please specify: ____________________

7. For how many days do you prescribe the treatment?

1) 7 days

2) 10 days

3) 14 days

4) Other; please specify: ____________________

8. What follow-up do you usually recommend after completing *H. pylori* treatment?

1) Do not refer to additional tests if symptoms resolved

2) Refer to specialist in gastroenterology at least 1 month after therapy

3) Refer the patient to urea breath test at least 1 month after therapy

4) Refer the patient to serology test at least 1 month after therapy

5) Refer to perform stool antigen enzyme immunoassay test at least 1 month after therapy

6) Other; please specify: ____________________

9. How do you usually do in patients with treatment failure after *H. pylori* treatment?

1) Do not refer to additional tests if symptoms resolved

2) Recommend the same treatment for a longer duration

3) Refer the patient to specialist in gastroenterology

4) Recommend a different treatment; please specify: ______________________

5) Other; please specify: ____________________

Module II: The following questions are related to the diagnosis and treatment of gastroesophageal reflux disease in adults.

1. Did you use any guidelines for the management of gastroesophageal reflux disease?

1) No

2) Yes, please specify: ____

4. How often do you choose each of the following for diagnosis and treatment in suspected cases of gastroesophageal reflux disease? (Please choose only one answer):

	Always	Usually, yes	Usually, no	Never
I refer to cardiologic work-up prior diagnosis of reflux, if complaints of chest pain exist	4	3	2	1
I refer to barium radiographs as part of a GERD diagnosis	4	3	2	1
I recommend weight loss for patients with overweight or obese as part of the treatment of uncomplicated GERD	4	3	2	1
I refer to evaluation of a specialist in gastroenterology in patients suspected to have GERD complications	4	3	2	1
I recommend empiric treatment with PPIs for patients with typical symptoms of uncomplicated GERD	4	3	2	1
I recommend to continue PPI therapy in patients who still suffer from GERD symptoms after cessation of initial treatment	4	3	2	1
I recommend on sleeping with elevated position as part of GERD treatment	4	3	2	1

I recommend changes in lifestyle as part of the treatment, such as reduced consumption of caffeine, chocolate and fried foods	4	3	2	1
